# Prediction of drug permeation through microneedled skin by machine learning

**DOI:** 10.1002/btm2.10512

**Published:** 2023-04-03

**Authors:** Yunong Yuan, Yiting Han, Chun Wei Yap, Jaspreet S. Kochhar, Hairui Li, Xiaoqiang Xiang, Lifeng Kang

**Affiliations:** ^1^ School of Pharmacy, Faculty of Medicine and Health University of Sydney New South Wales 2006 Australia; ^2^ Department of Clinical Pharmacy and Pharmacy Administration, School of Pharmacy Fudan University Shanghai 201203 China; ^3^ Harvard T.H. Chan School of Public Health 677 Huntington Avenue Boston Massachusetts 02115 USA; ^4^ National Healthcare Group 1 Fusionopolis Link Singapore 138542 Singapore; ^5^ Procter & Gamble 70 Biopolis Street Singapore 138547 Singapore; ^6^ MGI Tech 21 Biopolis Road, Nucleos Singapore 138567 Singapore

**Keywords:** machine learning, microneedle, multiple linear regression, random forest, transdermal, XGBoost

## Abstract

Stratum corneum is the outermost layer of the skin preventing external substances from entering human body. Microneedles (MNs) are sharp protrusions of a few hundred microns in length, which can penetrate the stratum corneum to facilitate drug permeation through skin. To determine the amount of drug delivered through skin, in vitro drug permeation testing is commonly used, but the testing is costly and time‐consuming. To address this issue, machine learning methods were employed to predict drug permeation through the skin, circumventing the need of conducting skin permeation experiments. By comparing the experimental data and simulated results, it was found extreme gradient boosting (XGBoost) was the best among the four simulation methods. It was also found that drug loading, permeation time, and MN surface area were critical parameters in the models. In conclusion, machine learning is useful to predict drug permeation profiles for MN‐facilitated transdermal drug delivery.

## 1 INTRODUCTION

A microneedle (MN) patch consists of an array of MNs of a few hundred microns in length, made of biocompatible materials, such as metals or polymers.^1^ These MNs can enhance drug permeation into the skin and/or main circulation, by creating multiple micron‐sized passages through skin, a formidable biological barrier preventing external substances from entering human body.^2^, ^3^, ^4^, ^5^ Owing to the simple concept of MN and technological feasibility of MN fabrication, MN has been extensively studied as a device to deliver into human a variety of therapeutic agents, for example, small‐molecule compounds,^6^, ^7^ peptides,^8^, ^9^, ^10^ mRNA,^11^ proteins,^12^ and extracellular vesicles.^13^

To determine the amount of drug entering human via skin, in vitro skin permeation testing can be performed. In such testings, the MN patch is mounted onto animal or human skin grafts in vitro, which are then placed in a Franz‐type diffusion device to monitor drug concentrations at different time points.^14^ The results from the tests can reveal the amount of drug penetrating skin as a function of time, which is critical to evaluate the performance of a MN patch, in terms of drug dose and therapeutic efficacy. However, it is costly and time‐consuming to perform such tests.^15^

To address this issue, simulation methods have been explored, that is, to predict drug permeation profiles using predictors, instead of conducting in vitro skin permeation experiments. In our previous study of a topical formulation, a multiple linear regression (MLR) model was constructed to link the permeability rate of the drug to the physicochemical properties of an excipient used in the formulation.^16^ With the established model, it was shown that the predicted values were good fits of the experimental results. The MLR model allows the prediction of the performance of the excipient, namely, skin penetration enhancers, without the need to conduct in vitro experiments using scarce human skin samples.

The prior study has motivated us to apply this method onto MN‐facilitated transdermal delivery, that is, to simulate the process and predict drug permeation through skin without performing in vitro skin permeation testing. Apart from the MLR method, additional machine learning methods were also investigated, because MN‐facilitated drug permeation profiles are more complex than those found in conventional topical dosage forms, hence advanced machine learning models may offer better predictions than the MLR model.

Machine learning is a subset of artificial intelligence, which builds a model based on the training data set to make predictions or decisions. Machine learning has been studied in various areas in medicine, such as cancer diagnostics, antibiotic synthesis, chemical reaction optimization, and protein structural prediction.^17^, ^18^, ^19^, ^20^, ^21^, ^22^, ^23^, ^24^, ^25^ In the field of drug delivery, it also found many applications. For example, a machine learning model was proposed to predict the potential drug–drug interactions, in terms of pharmacokinetic parameter changes of the patients.^26^ It can be used to evaluate drug–drug interactions before clinical trials, potentially useful for drug discovery and development.

Recently, machine learning has been used to optimize MN design and fabrication. For example, a deep learning (a subset of machine learning) method was used to aid the fabrication of MN patches. The model can be used to find efficient procedures for MN fabrication prior to actual production, saving materials and resources.^27^ In another example, drug diffusion from a polymeric MN patch was simulated, using surface erosion theory and Fick's law.^28^ The model allows users to optimize the MN design without fabricating the MN patch. Considering these recent developments, we hypothesize this approach can be used to predict drug permeation through skin.

In this study, machine learning was used to simulate the process of MN facilitated transdermal drug delivery and predict drug permeation through skin. The parameters in the models include MN features and the physicochemical properties of the drugs. The data points used in model training were collected from our previous studies on MN facilitated transdermal drug delivery, ensuring data consistency. Three machine learning methods were used, namely, first‐order MLR, random forest (RF), and extreme gradient boosting (XGBoost). In addition, a simulation method based on Fick's law was also included as a comparison. The key parameter used in Fick's law model was not obtained by using the training data set but from literature. To our knowledge, this is the first study to predict the drug permeation through MN‐treated skin using machine learning methods.

## 2 METHODS

### 2.1 Data collection

In our previous studies on MN‐facilitated transdermal drug delivery, we performed in vitro skin permeation experiments using similar protocols and devices for six different drugs/chemicals, namely, caffeine, copper, peptide, bovine serum albumin (BSA), lidocaine, and rhodamine. During the experiments, the time course of drug permeation through skin was determined by using Franz‐type of diffusion devices. The data points from those studies were compiled into a single database.

For copper^9^ and peptide,^9^ the data are based on plastic MNs, obtained by Li et al. For caffeine,^29^ BSA,^30^ and rhodamine,^31^ the skin permeation data are based on hydrogel MNs, obtained by Neo and Kochhar et al. For lidocaine, the skin permeation data are based on both plastic MNs and hydrogel MNs, obtained by Kochhar^12^ and Li et al.^32^ The whole data set can be found in Data S1.

### 2.2 Overview of the simulation methods

**FIGURE 1:**
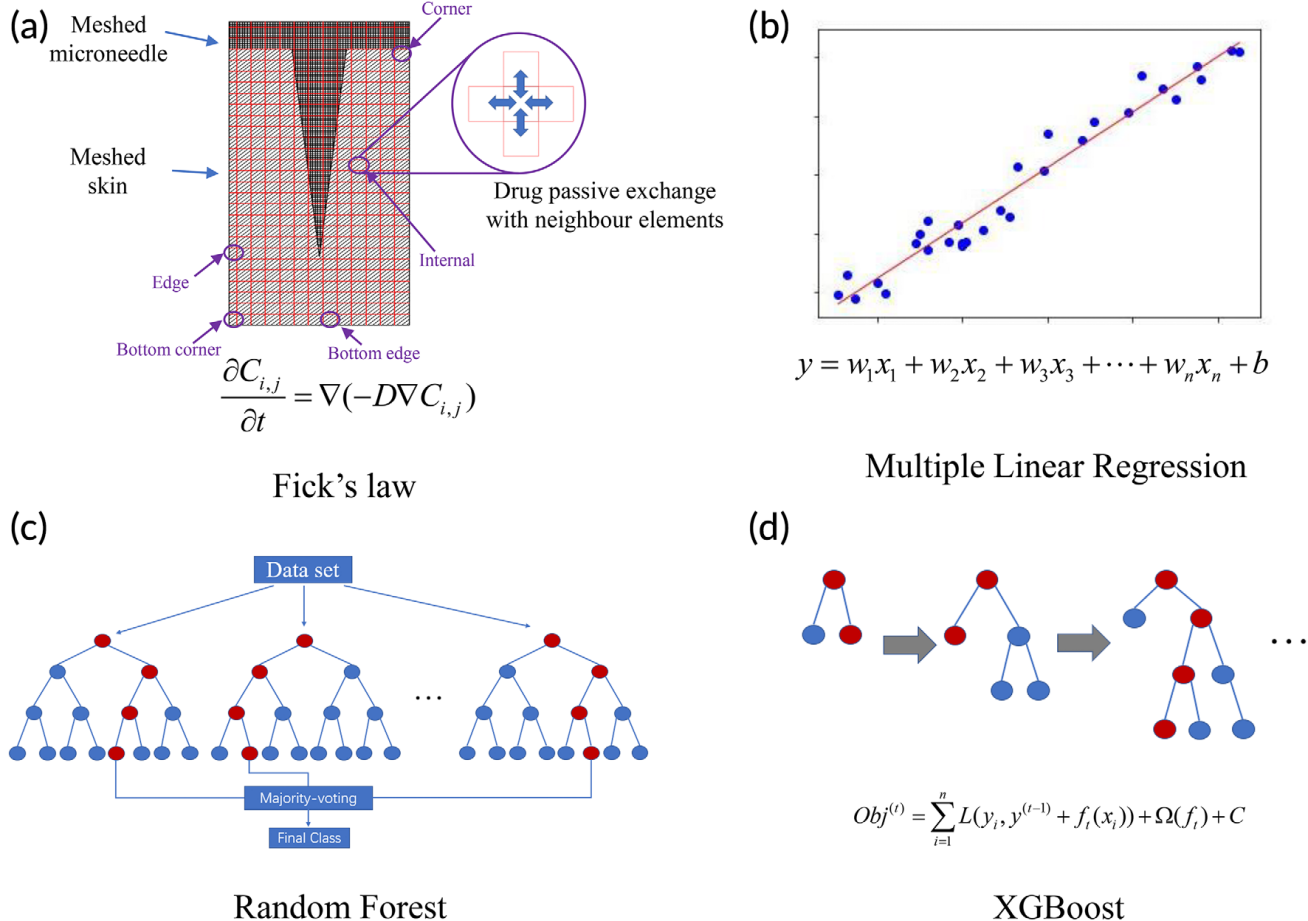
Four methods were used to predict drug permeation through skin: (a) Fick's law; (b) multiple linear regression; (c) random forest; and (d) XGBoost.

The four methods are illustrated in Figure 1. For Fick's law, a two‐dimensional model representing the central cross‐section of the MN and skin was used to predict drug permeation through skin, as shown in Figure 1a. The diagram illustrates a typical setup used for in vitro skin permeation testing, where the MN was applied onto skin. The drugs encapsulated in the MN will diffuse into the skin tissue and in turn diffuse into the receptor solution. The drug concentration inside the receptor solution was measured at different time points to obtain the cumulative amount of drug permeated through skin.

For all four methods, the dependent variable is the cumulative percentage/amount of drug permeated through skin. The descriptors, such as permeation time and MN features, were used to predict the percentage/amount of drug permeated through skin. In the first order MLR model, the dependent variable was assumed to be linearly correlated to the descriptors, as shown in Figure 1b. The linear assumption may not be valid, but MLR can provide a simple model for prediction. The RF and XGBoost methods are based on the decision trees, as shown in Figure 1c,d.

The data points collected from the in vitro skin permeation testing were randomly divided into the training set and test set. The training set was used to build the models in the machine learning algorithm, while the test set was used to verify the robustness and precision of the models, namely, MRL, RF, and XGBoost.

### 2.3 Fick's law

During the process of drug permeation through skin, the drug concentration in the receptor compartment of the Franz diffusion device can be calculated by using Fick's second law of diffusion, as shown in Equation (1):

(1) $$\frac{\partial C}{\partial t}=D\left(\frac{\partial^{2}C}{\partial x^{2}}+\frac{\partial^{2}C}{\partial y^{2}}\right).$$

In Equation (1), C is the concentration of the drug at time t while D is the diffusion coefficient. With initial and boundary conditions given, as those in skin permeation experiments, an analytical solution can be obtained from Equation (1), for non‐MN facilitated drug permeation through skin.^33^, ^34^ However, the drug permeation process becomes complex when MNs were inserted into skin membrane. In this case, it is challenging and/or unnecessary to obtain an analytical solution, as drug concentrations inside skin depend not only on skin depth but also the geometry of the MNs. To this end, numerical solutions based on domain discretization can be useful, as they approximate the differential equation's solution and calculate each subdomain rather than over the entire domain. Recently a study reported numerical solutions for MN‐facilitated drug permeation through skin method, using a commercial software (COMSOL Multiphysics).^35^ In this study, we use C language to write the model to obtain a numerical solution to Equation (1) for MN‐facilitated drug permeation.

To simplify the modeling process, we used a two‐dimensional model to represent the 3D MN‐skin system, as shown Figure 1a. The 2D model shows the cross‐section of the MN and the total amount of drug in the 3D MN is evenly distributed on the 2D surface. An MN patch normally consists of multiple identical MNs with fixed spacing between each MN. For modeling purpose, we assume that each MN‐skin unit, as shown in Figure 1a, is independent of other MN‐skin units in the mesh and considered as a repeating unit. Hence, there are five types of mesh elements, namely, corner, bottom corner, edge, bottom edge, and internal elements, as shown in Figure 1a. For each type of element, their boundary conditions are different. For instance, the internal element shown in Figure 1a will exchange the drug with its top, down, left, and right elements while the edge element will exchange the drug with its top, down, and right element, but there is no left element to exchange with. The exchange of drug can be calculated with concentration gradient multiplied by diffusion coefficient.

Based on this model, the left part of Equation (1) represents the drug concentration in an arbitrary element varied with time. The right part of Equation (1) represents the drug exchange of that element with its neighbor elements. Equation (1) can be solved by using Taylor's formula to calculate drug concentration in each element.^36^ The drug concentration in each element of whole meshed MN‐skin system can be updated with every time step. Once the drug molecules gradually permeate through skin and reach the receptor liquid, the amount of drug in the receptor, at a certain time point, can be calculated by multiplying the volume of the receptor solution with the drug concentration at that time point. Afterwards, the total amount of drug in the receptor solution can be obtained by multiplying the amount of drug from one MN with the total number of MNs on one MN patch.

The cumulative amount of permeated drug was defined as the amount of drug through an area of 1 cm^2^, which refers to the diffusion window between the donor compartment and receptor compartment in the diffusion device. In our previous in vitro skin permeation experiments, two types of Franz diffusion devices were used, that is, vertical diffusion device and horizontal diffusion device. In the vertical diffusion device,^9^ the diffusion area is 1 cm^2^ and the horizontal diffusion area^12^ is 1.13 cm^2^. So, the experimental cumulative amount of drug was normalized to be through an area of 1 cm^2^.

The cumulative drug permeation percentage is calculated by the cumulative amount of permeated drug from time zero to *t* ($m_{t}$), divided by the total amount of drugs impregnated inside the MN ($m_{\text{total}}$), as shown in Equation (2):

(2) $$\text{Permeation percentage}=\frac{m_{t}}{m_{\text{total}}}\times 100\%.$$

In this model, five assumptions were made to simplify the modeling process. First, the shape of the MN is incompressible. The MN is seen as nondegradable and not compressible after it is inserted into skin. Second, the drugs in MN are uniformly distributed. Moreover, the drug concentration in each element is uniform. The drug diffuses in *X* and *Y* directions only, as this is a 2D model. Third, the diffusion coefficient of the drug is constant in skin, and the temperature effect is neglectable, as the skin permeation study is normally held at human body temperature. Fourth, the permeated drugs will not block the interface between skin and the receptor solution in the Franz device. The drugs will be instantly distributed to the receptor solution after they penetrate the skin membrane, as stirring is always provided in the receptor compartment in our drug permeation experiments. Fifth, the skin thickness is assumed to be 1 mm for all experiments. This is reasonable as the MN is of a few hundred microns in length while the skin membrane is ~1 mm thick.

### 2.4 Multiple linear regression (MLR)

MLR is a regression model estimating a dependent variable based on multiple independent variables (Figure 1b). For a simple linear regression model, the formulation includes a response (y) and one variable (x), a slope (k) and an intercept (b), as shown in Equation (3). MLR extends one variable in linear regression to multiple variables. All variables contribute to the response. The formula of MLR is given in Equation (4).

(3) y=kx+b,

(4) $$y=k_{1}x_{1}+k_{2}x_{2}+\cdots +k_{n}x_{x}+b.$$

### 2.5 Random forest (RF)

RF is based on the decision tree method, as shown in Figure 1c. The RF model consists of multiple trees, and each tree is independent of other trees. A decision tree starts to build from the root node to the sub‐nodes until it meets the maximum layer, or no features can be used. During the process of construction of a decision tree, several critical experimental features are selected by the model. This process is repeated several times, producing multiple trees. The output result of an RF model is the majority voting for classification or the average value for regression. The contribution of each feature to the RF model is different. The greater the importance of a feature, the more frequently it would be selected by the decision trees in the RF model.

### 2.6 Extreme gradient boosting (XGBoost)

The XGBoost is also based on the decision tree method, as shown in Figure 1d. However, unlike the RF, the trees in XGBoost are related to one another. The former tree results are used to guide the development of the next tree. The objective formula in the XGBoost is shown in Equation (5):

(5) $$\mathrm{Obj}=\sum_{i=1}^{n}l\left(y_{i},{\hat{y}}_{i}\right)+\sum_{k=1}^{K}\Omega \left(f_{k}\right).$$

The objective formula is made up of two parts, the first part $\sum_{i=1}^{n}l\left(y_{i},{\hat{y}}_{i}\right)$ is the training loss, and the second part $\sum_{k=1}^{K}\Omega \left(f_{k}\right)$ represents the complexity of the trees. The $\Omega \left(f_{k}\right)$ could be expressed as Equation (6):

(6) $$\Omega \left(f_{k}\right)=\mathrm{\gamma T}+\frac{1}{2}\lambda \sum_{j=1}^{T}w_{j}^{2}.$$

In Equation (6), γ represents the minimum loss reduction required to make a further partition on a leaf node of the tree, T the number of the leaves, w the score on the leaf, and λ is the weight for w. Adjusting the complexity of the trees could reduce model overfitting when trying to minimize the objective formula.

### 2.7 Model evaluation

The predicted results and the actual experimental data were compared using two parameters, that is, the root mean square error (RMSE) and *R*‐squared (*R* ^2^), as shown in Equations (7) and (8).

(7) $$\text{RMSE}=\sqrt{\frac{1}{n}\sum_{i=1}^{n}{\left(y_{i}-{\hat{y}}_{i}\right)}^{2}},$$

(8) $$R^{2}=1-\frac{\sum_{i}^{n}{\left({\hat{y}}_{i}-y_{i}\right)}^{2}}{\sum_{i=1}^{n}{\left({\bar{y}}_{i}-y_{i}\right)}^{2}}.$$

The smaller the RMSE value, the better the prediction is. For *R* ^2^, a value closer to 1 indicates a better prediction.

### 2.8 Simulation process

**FIGURE 2:**
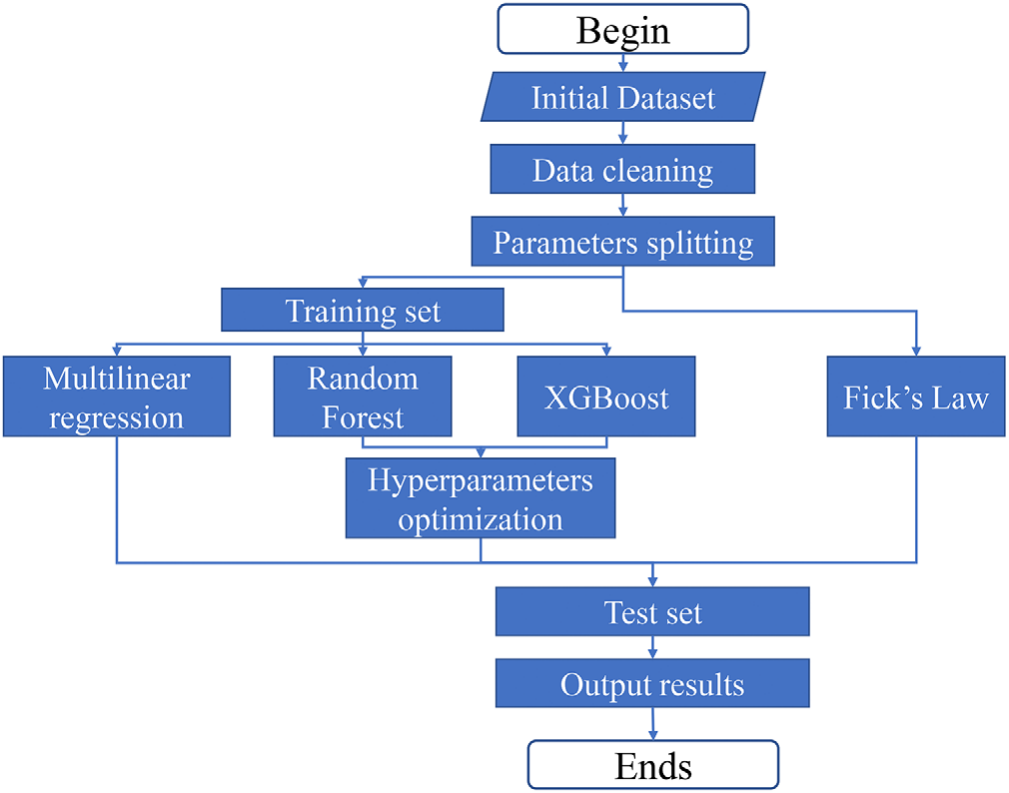
A flowchart showing the model training and prediction process of all four models: Fick's law, MLR, RF, and XGBoost. MLR, multiple linear regression; RF, random forest.

The simulation process is illustrated in Figure 2. After the manual data cleaning process, a data set containing 191 experimental data points was obtained, as shown in Data S1. The 191 data points were randomly split into training set and test set, at a ratio of 7:3. Afterwards, the models were subjected to the training process to obtain the final models. The model based on Fick's law did not undergo the training process, as the key parameter (diffusion coefficient) for each drug was found in literature. After the four models were obtained, the data points in the test set were used to verify the accuracy of the predicted numerical values. The prediction accuracy of the four methods, that is, how close the simulated results to the experimental data, was compared using both RMSE and *R* ^2^.

The codes of MLR, XGBoost, and RF were written with the R language (R version 4.1.2) in R Studio. The version of XGBoost and RF were 1.5.0.2 and 4.7.1, respectively. The code of Fick's law was written using the C language in Microsoft Visual Studio 2022. The codes of all four methods can be found in Data S2.

## 3 RESULTS

### 3.1 Data collection

**FIGURE 3:**
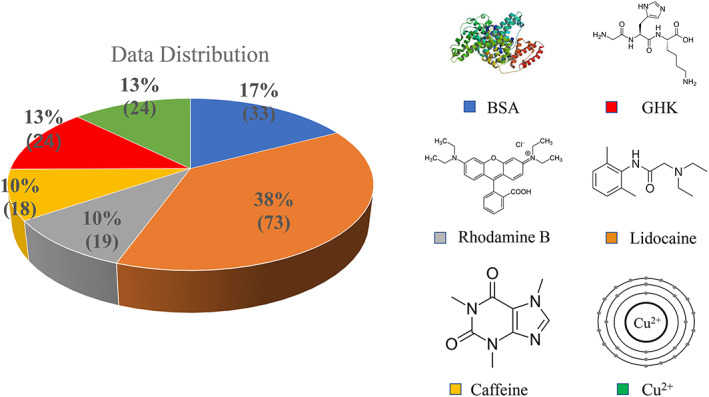
The data distribution and molecular structure of the drugs delivered using MNs in the skin permeation study.^9^, ^12^, ^30^, ^31^ MNs, microneedles.

The experimental data consist of six different drugs, namely, BSA, copper ions, a tripeptide glycyl‐l‐histidyl‐l‐lysine (GHK), rhodamine B, lidocaine, and caffeine, as shown in Data S1. Compared with others in this study, BSA is a relatively large molecule, with a molecular weight (MW) of 66,000.^30^ GHK is a naturally occurring carrier tripeptide with high affinity for copper ions.^9^ The total number of data points is 191, of which each drug contributes at a percentage as shown in Figure 3.

In the data set, lidocaine has 73 data points, accounting for 38% of all data points. Followed are BSA (33%), GHK (24%), and copper (24%). Lastly, rhodamine B and caffeine have relatively smaller number of data points, accounting for 10% each. There are four small molecules (GHK, rhodamine, lidocaine, caffeine), one metal ion (copper), and one big molecule (BSA), representing different classes of permeants, with the majority being small molecules. Overall, the total number of data points is small, which is a limitation of this study.

### 3.2 Fick's law

The parameters used in Fick's law are listed in Table 1. The diffusion coefficient of each drug was found in literature.^37^, ^38^, ^39^, ^40^

**TABLE 1:** The parameters used in Fick's law simulation.^9^, ^12^, ^30^, ^31^

Parameters	Values
Diffusion coefficient (*D*)	50–1000 μm^2^/min^37^, ^38^, ^39^, ^40^
MN number (*N*)	64, 351
Microneedle length (*L*)	700, 820, 876, 889, 999, 1250, 1063 μm
Releasing time (*t*)	15 min–48 hr
Mass of loaded drug (*m*)	50–70,940 μg
Grid size (*dx*, *dy*)	2 × 2
Time step (dt)	0.00001–0.001 min

**FIGURE 4:**
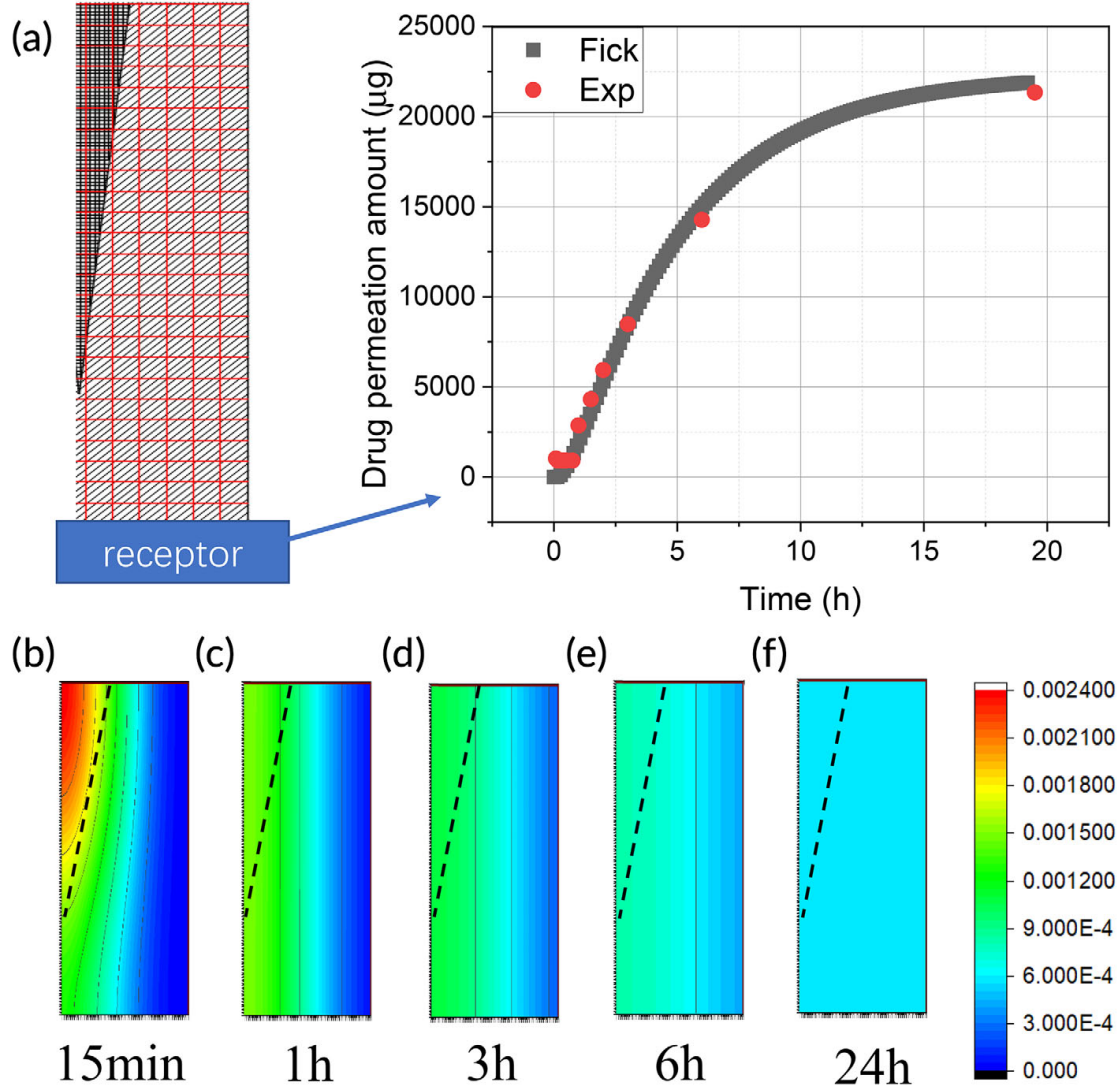
(a) An illustration of the Fick's law prediction curve compared with the experimental data. The drug distribution in the MN‐skin system at (b) 15 min, (c) 1 h, (d) 3 h, (e) 6 h, and (f) 24 h. MN, microneedle.

The left panel of Figure 4a shows the setup of a typical in vitro skin permeation experiment, with the MN being inserted into skin membrane, which was then placed above a receptor compartment (blue color block) filled with receptor solution, collecting the drug molecules permeated through skin. The upper left section represents the half MN, and the lower right section represents the adjacent skin tissue. The bottom area represents the receptor compartment of a Franz type diffusion cell. Since the two‐dimensional model is symmetric, only half of the model is used in calculation to save computer resources and time.^41^

On the right panel of Figure 4a, the predicted data points (black dots/line), and the experimental data points (red dots) of lidocaine permeation through skin are plotted together. At the beginning part of curve, the drug permeation rate increases, shown by the gradually increasing slope. This is resulted from the drugs released from the MNs, which then permeated into the skin tissue adjacent to the MN, as shown in Figure 4b. In the heat map, the red color indicates high drug concentration inside the MN while the green/blue color indicates the lower concentration of the drug. The dash lines in Figure 4b–f distinguish between the MN section at the upper left and the skin tissue at the lower right.

As the drug permeation from MN to skin continues, more drug molecules from the MN patch diffuse into the skin tissue gradually, as shown in Figure 4c–f. As a result, the rate of drug permeation from skin tissue into the receptor solution continues to increase, as depicted in the middle part of the curve. As the diffusion process continues, more drug molecules diffuse into the receptor solution.

After a period, the drug permeation reached a critical point where the permeation rate decreased and plateaued eventually. This is because the drug concentration in the MN, the skin and the receptor solution reached an equilibrium, such as that drug diffusion from MN to skin, or from skin to receptor solution stopped, as shown in Figure 4f.

The simulated curve fits well with the experimental points, demonstrating that the MN‐facilitated drug permeation process complied with Fick's law of diffusion, that is, the MN provide a finite amount of drug reservoir and the drug continues to diffuse into skin till the concentration gradient ceases to exist.

Although the diffusion model is based on the hydrogel type of MN, that is, the MNs were inserted inside skin during the drug permeation process, the diffusion model was also used for the plastic type of MN. For the plastic MNs, the skin is pre‐treated with the plastic MN patch and left with an array of micron‐sized passages, complementary to the contour of the plastic MN.^9^ The pre‐treated skin sample is then mounted between the donor and receptor compartment of Franz‐type diffusion device for drug permeation testing. The aqueous solution containing the drug in the donor compartment will fill in the micron‐sized passages, to further permeate into skin tissue and subsequently the receptor compartment, resembling that of the hydrogel type of MN.

For the hydrogel MNs, the drug loading is defined as the amount of drug impregnated inside the MN patch. For plastic MNs, the drug loading is defined as the amount of drug, which is dissolved in an aqueous solution in advance, added to the donor cell in the in vitro skin permeation experiments. During the drug permeation process, the drug concentration was assumed to be homogenous inside the donor compartment and the micron‐sized passages inside skin tissue. As the permeation process continues, the drug concentration inside the donor compartment and micron‐sized passages decreases, which also resembles that of the hydrogel MN patch.

Based on these assumptions and approximations, we used a single mathematical model to simulate both type of MN patches for drug permeation through skin membranes. The data from in vitro permeation experiments were fitted using the model, regardless of hydrogel or plastic MN patches.

**FIGURE 5:**
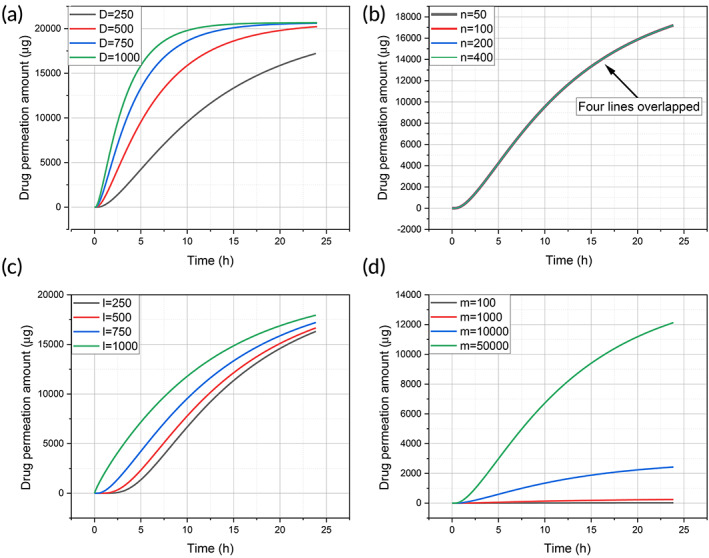
Effect of different parameters in Fick's law simulation: (a) diffusion coefficient; (b) number of MN; (c) MN length, and (d) mass of loaded drugs. MN, microneedle.

For the Fick's law model, the effect of the key parameters, namely, diffusion coefficient, the number of MNs, the length of MN, and drug loading inside the MN, on drug permeation through skin are shown in Figure 5.

The greater the diffusion coefficient, the quicker the drug permeation, as shown in Figure 5a. When the diffusion coefficient increases from 250 to 1000 μm^2^/min, the drug permeation increased accordingly. Figure 5b illustrates the effect of the number of MN on a MN patch. It was shown that the number of MN had no effect on the drug permeation profile, when the amounts of loaded drugs in the MN patch were the same. Therefore, all four lines overlapped to become a single curve in Figure 5b. Next, longer MN can deliver drugs into deeper skin, hence increase drug permeation, as shown in Figure 5c. Lastly, increasing the amount of drug loading results in greater drug permeation, as shown in Figure 5d. Drug loading has a greater impact on drug permeation than MN length.

### 3.3 Machine learning

To build the machine learning models, seven features were selected for MLR, RF, and XGBoost, including the skin type (rat and human), type of the MN (hydrogel and plastic), MN length, surface area of the MN, drug loading in MN, drug permeation time, and the MW of the drug. Skin type,^9^ drug permeation time,^33^ and drug MW^42^ are found affecting drug permeation through skin in our previous studies on non‐MN facilitated transdermal drug delivery. The type of MN, MN length, surface area of MN, and drug loading in MN are potentially important parameters we identified for MN‐facilitated transdermal drug delivery, hence included in the models in the current study.

Two types of MNs were used in the in vitro skin permeation experiments we performed previously. The first type of MN is made of a biocompatible polymer, namely, polyethylene glycol diacrylate (PEGDA), impregnated with drugs, in the shape of a frustum.^31^ Upon application into skin, the PEGDA MNs will absorb moisture to form a hydrogel, and in turn release drugs into skin tissue. So, it is named as hydrogel type of MN. The other type of MN is made of plastics, in the shape of a pyramid.^9^ The plastic MN is used to pre‐treat skin membrane to increase skin permeability, and then removed from the skin membrane. The treated skin membrane was then mounted onto the drug permeation testing device and a small volume of aqueous solution of the drug was added into the donor compartment of the testing device where drug permeates through the pre‐treated skin membrane.

MN surface area is an important parameter, as it represents the total contact surface area of a MN patch with skin tissue, where drug permeates into skin tissue. For the hydrogel MNs, it is approximately in the shape of a frustum, like a truncated cone. For the surface area of hydrogel MN, it is calculated using Equation (9), where S is the surface area of single MN, r the radius of top circle, R the radius of bottom circle, and l is the slant height of the conical frustum.

(9) $$S=\pi r^{2}+\pi \left(\mathrm{Rl}+\mathrm{rl}\right).$$

For the plastic MN, each MN is in the shape of a regular square pyramid. So, the surface area is calculated using Equation (10), where a is the length of the bottom square edge, and h is the height of a single MN from the apex of the pyramid to the base plane.

(10) $$S=4\cdot \frac{1}{2}a\cdot \sqrt{{\left(\frac{a}{2}\right)}^{2}+h^{2}}.$$

The total surface area of a MN patch is calculated using Equation (11), where $S_{\text{total}}$ is the total surface area of all MNs on one MN patch, and n is the total number of MNs integrated on a single MN patch.

(11) $$S_{\text{total}}=S\cdot n.$$

For example, the length of the bottom square edge of a plastic MN is 0.075 mm, and the height of a single MN is 0.7 mm. So, for a plastic MN patch consisting of 351 pieces of single MNs,^9^ the total MN surface area is:

$$S=4\cdot \frac{1}{2}a\cdot \sqrt{{\left(\frac{a}{2}\right)}^{2}+h^{2}}=4\cdot \frac{1}{2}\cdot 0.075\cdot \sqrt{{\left(\frac{0.075}{2}\right)}^{2}+0.7^{2}}=0.105\;{\mathrm{mm}}^{2},$$

$$S_{\text{total}}=S\cdot n=0.105\times 351=36.855\;{\mathrm{mm}}^{2}.$$

The MW of drug may also influence the drug permeation and it was included as well. The features and their values are listed in Table 2. The hyperparameters used in RF and XGBoost models for drug permeation amount and percentage are shown in Table 3.

**TABLE 2:** The parameters used in MLR, RF, and XGBoost.^9^, ^12^, ^30^, ^31^

Feature	Value
Skin type	Rat (R), human (H)
MN type	Hydrogel, solid
MN length (μm)	700, 820, 889, 1250, 875.97, 998.62, 1062.97
MN surface area (mm^2^)	26.76, 28.54, 29.97, 32.13, 32.43, 34.49, 36.86
Drug loading (μg)	50–70,940
Permeation time (hr)	0.08333–48
MW (Da)	64, 194, 234, 340, 479, 66,430
Drug permeation (μg)	0–30,000

Abbreviations: MLR, multiple linear regression; MN, microneedle; MW, molecular weight; RF, random forest.

**TABLE 3:** The hyperparameters used in the RF and XGBoost.

XGBoost parameter	Value	RF parameter	Value
Max depth^a^	4	Trees number^a^	500
Eta^a^	0.4	Mtry^a^	5
Nround^a^	100		
Max depth^b^	3	Trees number^b^	500
Eta^b^	0.2	Mtry^b^	6
Nround^b^	45		

^a^ Drug permeation amount prediction.

^b^ Drug permeation percentage prediction.

### 3.4 Method comparison

To compare the four simulation methods, two parameters were used, that is, the RMSE and *R* ^2^, the definitions of which are shown in Equations (7) and (8). For RMSE, it is the root of summation of all the squared difference of the predicted result and the experimental result, divided by the number of data points. So, a smaller RMSE value indicates a better prediction, that is, the predicted results are closer to the experimental results. Although easy to understand and interpret, the numerical value of RMSE depends on the unit of the data points. Different from RMSE, *R* ^2^ does not depend on the unit of the data, hence useful to make comparisons with studies using different units.

The RMSE and *R* ^2^ results of four simulation methods are shown in Table 4. XGBoost had the lowest RMSE value and highest *R* ^2^ for both permeation amount and percentage prediction, among the four simulation methods. Between RF and Fick's law, RF showed better performance in permeation percentage prediction, while Fick's law was better in predicting permeation amount. MLR showed the poorest prediction among the four methods for both amount and percentage permeation.

**TABLE 4:** Comparison of the four simulation methods.

RMSE and *R* ^2^	XGBoost	RF	Fick's law	MLR
RMSE of permeation amount (μg)	4447.23	7043.97	6778.17	23398.91
*R* ^2^ of permeation amount	0.98	0.95	0.95	0.46
RMSE of permeation percentage (%)	28.24	34.33	85.58	120.33
*R* ^2^ of permeation percentage	0.98	0.97	0.82	0.65

Abbreviations: MLR, multiple linear regression; RF, random forest; RMSE, root mean square error.

**FIGURE 6:**
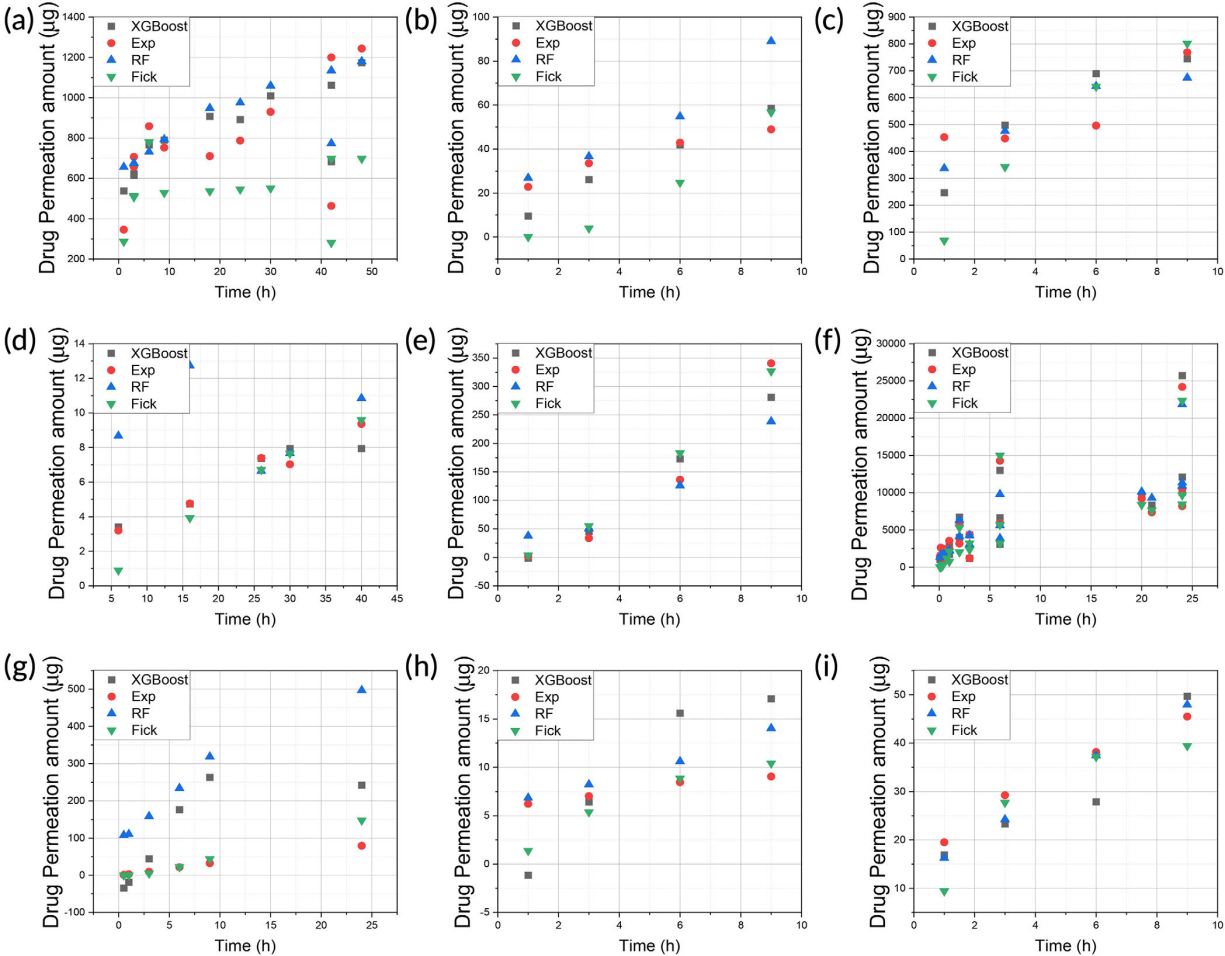
The comparison of the predicted permeation results with experimental data: (a) BSA(R); (b) GHK(H); (c) GHK(R); (d) rhodamine B(R); (e) lidocaine(H); (f) lidocaine(R); (g) caffeine(H); (h) Cu(R); and (i) Cu(H) (R: rat skin; H: human skin). BSA, bovine serum albumin; GHK, glycyl‐l‐histidyl‐l‐lysine.

**FIGURE 7:**
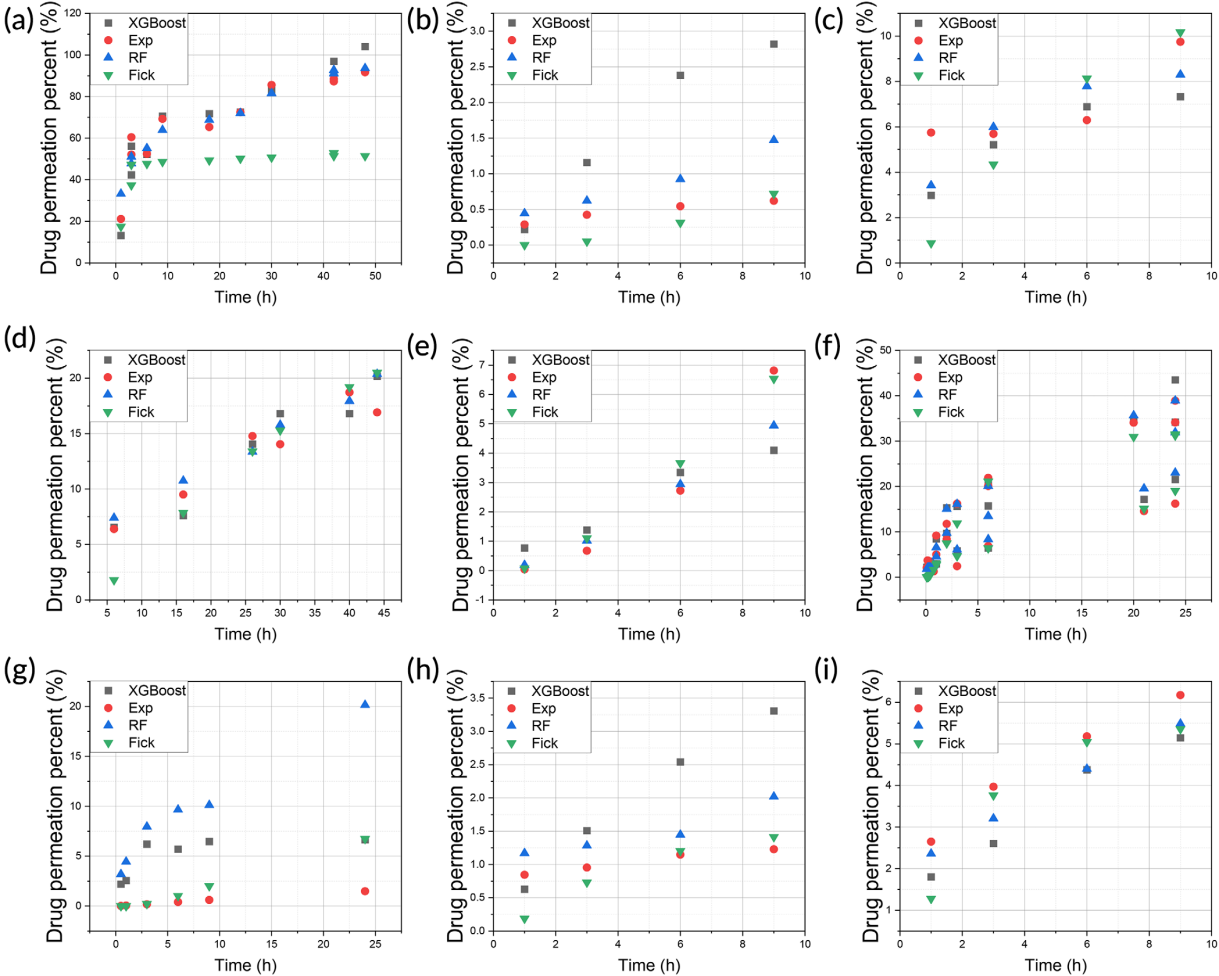
The comparison of the predicted drug permeation percentage with experimental data: (a) BSA; (b) GHK(H); (c) GHK(R); (d) rhodamine B(R); (e) lidocaine(H); (f) lidocaine(R); (g) caffeine(H); (h) Cu(R); and (i) Cu(H) (R: rat skin and H: human skin). BSA, bovine serum albumin; GHK, glycyl‐l‐histidyl‐l‐lysine.

The comparison of predicted results against experimental data was shown in Figure 6 (drug permeation amount) and Figure 7 (drug permeation percentage), respectively. The MLR result was excluded to reduce overcrowding in the figures as it has the poorest prediction. There are six drugs permeating through human and/or rat skin, namely, BSA through rat skin (Figure 6a and Figure 7a), GHK through human skin (Figure 6b and Figure 7b), GHK through rat skin (Figure 6c and Figure 7c), rhodamine B through rat skin (Figure 6d and Figure 7d), lidocaine through human skin (Figure 6e and Figure 7e), lidocaine through rat skin (Figure 6f and Figure 7f), caffeine through human skin (Figure 6g and Figure 7g), copper through rat skin (Figure 6h and Figure 7h), and copper through human skin (Figure 6i and Figure 7i).

In each figure, the experimental points were color coded in red, while the Fick diffusion predictions were coded green, RF predictions were coded blue, and XGBoost predictions were coded black. The *x*‐axis represents the permeation time in hours while the *y*‐axis represents either the drug permeation amount (μg) (Figure 6) or permeation percentage (Figure 7). When the predicted result is close to the experimental point, the prediction is deemed to be better than when the predicted result is far from the experimental point. The quantified prediction results, that is, RMSE and *R* ^2^ were shown in Table 4.

For permeation amount prediction, as shown in Figure 6, the XGBoost and RF predictions matched well with experimental data points. However, there were significant deviations, when the data set was relatively small for the drug, namely, rhodamine B and caffeine, as shown in Figure 6d,g. For rhodamine B or caffeine, each drug has only less than 20 data points, accounting for only 10% of the data (Figure 3). For the permeation percentage prediction, as shown in Figure 7, the results were similar to those of permeation amount prediction, as shown in Figure 6.

### 3.5 Feature significance

**FIGURE 8:**
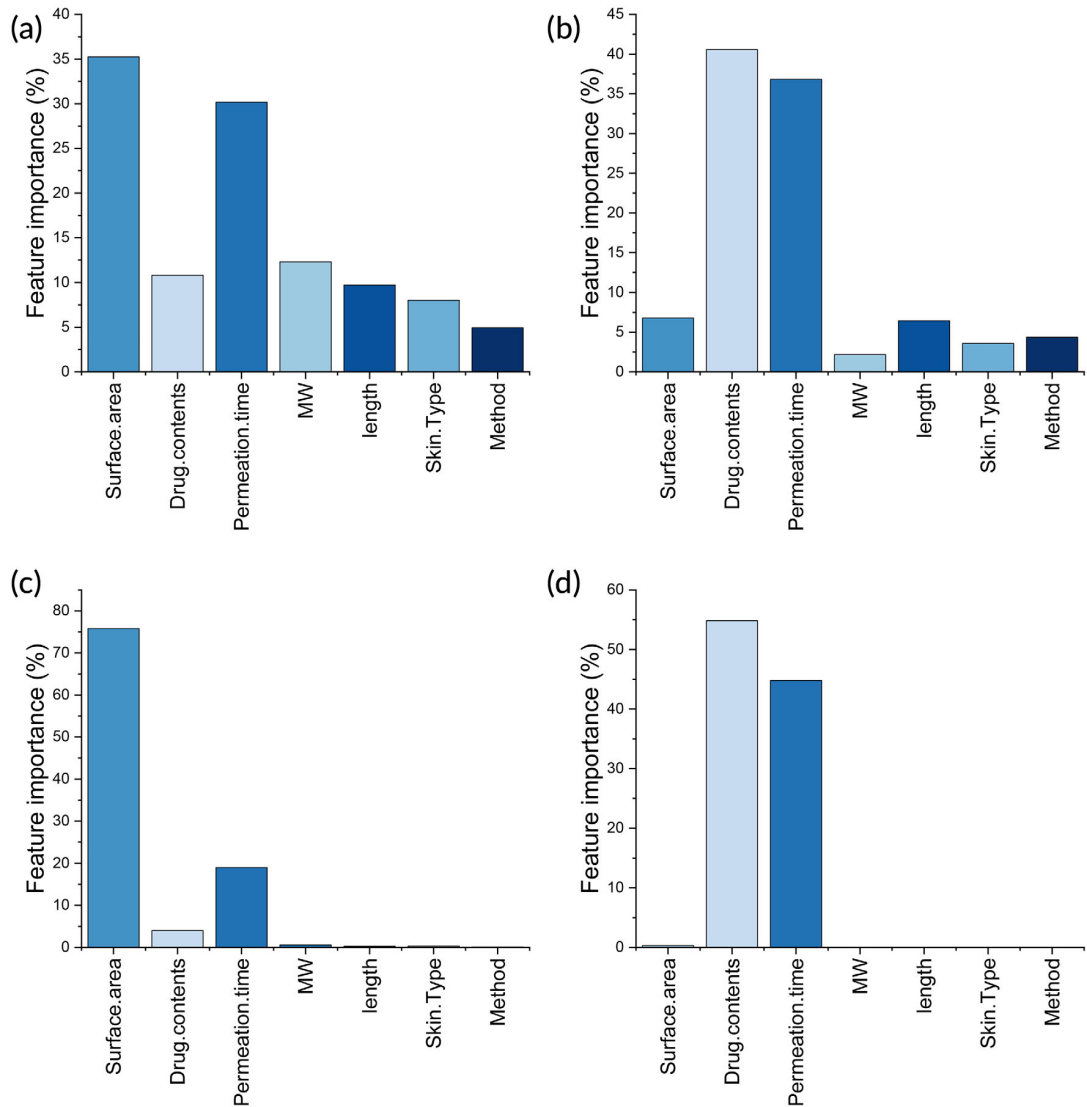
The feature significance in the RF and XGBoost models: (a) permeation percentage (RF); (b) permeation amount (RF); (c) permeation percentage (XGBoost); (d) permeation amount (XGBoost). RF, random forest.

The importance of different features used in the model in predicting drug permeation is shown in Figure 8. For RF and XGBoost, the surface area of the MN and permeation time were shown to be the key features in predicting drug permeation percentage (Figure 8a,c), while the drug loading in MN and permeation time were the key features in predicting drug permeation amount (Figure 8b,d). Hence, permeation time is a common feature for both drug permeation amount and permeation percentage prediction, which is consistent with Fick's law of diffusion. For permeation percentage, surface area of MN was found important, meaning that larger surface can result in a higher percentage drug loaded in the MN patch to be delivered into human. For permeation amount, drug loading was found important, meaning that higher drug loading can result in greater amount of drug to be delivered into human. Although both surface area and drug loading are important for drug permeation through skin, their contributions to drug permeation percentage and amount are different, as shown by the modeling results.

Besides, for RF method, the importance of drug MW, drug content, MN length, skin type, and MN type were below 12%, in predicting drug permeation percentage. The importance of MN surface area, MN length and type, skin type, and drug MW were below 7% in predicting permeation amount. These features were included in RF but have limited influence on the prediction. In XGBoost, drug content had less than 3% contribution in predicting drug permeation percentage. All other features showed little relevance in the prediction.

## 4 DISCUSSION

### 4.1 Mechanistic and statistical modeling

In our previous studies, we used mechanistic modeling to predict crystal growth of materials for additive manufacturing,^41^ to predict mammalian cell seeding inside microwells for tissue engineering,^43^ and to count cells automatically on microscopic images for image recognition applications.^44^ These methods are based on physical rules, such as the heat transfer equation and Stokes' formula of falling velocity. For such mechanistic models, the parameters in the models were obtained from the properties of the objects under investigations, for example, material crystallinity and dimension of the cells. Apart from the mechanistic models, we also studied statistical models, such as MLR, to make inferences and predictions of drug permeation through skin.^16^ For statistical models, the models are not based on physical rules and the parameters in the models were obtained from experimental data, not based on the objects themselves (materials, cells, microwells, etc.).

In the current study, we used both mechanistic models (Fick's law of diffusion) and statistical models (MLR, RF, and XGboost) to predict drug permeation through MN‐treated skin membrane. In comparison with MLR, RF and XGboost are more precisely defined as machine learning methods, which are focused on prediction than inference, but closely related to statistical models.^45^ One of the characteristics of machine learning is that the data set is divided into training set to obtain the model, and test set to validate the model. The other characteristic of machine learning is that larger data set is needed to obtain satisfactory out‐of‐sample predictions. In this study, we regard all three statistical models as machine learning models.

All four methods in this study are to predict the drug permeation through the skin, in terms of the amount (μg) of drug and as a percentage of total amount of drug loaded in the MN. Among the four methods, RF is a widely used algorithm, which grows a series of decision trees for regression and classification. XGBoost is also based on tree model, but it provides parallel tree boosting and is the leading machine learning library for regression, classification, and ranking problems. Among the four methods, XGBoost showed the best performance, for predicting both the drug permeation amount and percentage.

### 4.2 Model comparison

Fick's law is useful to understand the underlying mechanism of how drugs are released from MN and further permeate through the skin.^46^, ^47^, ^48^ The drug diffusion process can be calculated at any specified time according to Fick's law (Figure 4). However, one of the key parameters, namely, the diffusion coefficient is not readily available.^37^ In addition, diffusion coefficient is dependent on various factors, such as the depth of the skin, ambient temperature, and skin type.^28^, ^35^ This is the reason why machine learning methods are advantageous, since diffusion coefficient is not needed in machine learning methods, as demonstrated by the MLR, RF, and XGBoost models.

Although XGBoost produced the best predictions, the data sets were relatively small for caffeine and copper peptide, which affected the prediction precision to some extent (Figure 6d,g and Figure 7d,g). To further validate the XGBoost model, the model was used to predict the permeation of a drug which was purposely removed from the training data set. However, a large deviation was observed for both permeation amount (Figure S1) and percentage (Figure S2). The deviation may be resulted from the wide distribution of drug loading from various drugs used in the study, which is a heavily weighted parameter in XGBoost model. As a result, the predicted result for the new drug was dictated by drug loading. To address this issue, more experimental points should be obtained to cover the full range of drug loading (50–70,940 μg) for each drug in the training set in future studies. Besides, more experimental data are needed to increase the weight of the other parameters, such as MW of different drugs, number of needles per MN patch, and needle spacing on a MN patch. This can be achieved by increasing the number of drug candidates, which are then delivered using MN patches of different designs.

Nonetheless, the XGBoost can provide predictions if the drug is included in the training set, which is useful to evaluate the drug permeation profiles of varying MN patch designs. For example, the drug permeation profile of an MN patch with a hypothetical drug loading and surface area can be predicted by using the trained XGBoost model. This can aid the design of MN patches, without performing actual in vitro skin permeation experiments. Currently many MN devices for drug delivery are under clinical trial, for example, MN‐facilitated transdermal delivery of zolmitriptan, a painkiller to treat migraine.^49^ In such cases, the machine learning method may be used to generate optimal MN patch design to deliver certain doses of drugs into human, accelerating the development process of new therapeutics.

### 4.3 Feature comparison

The contribution of different features used in the model was shown in Figure 8. Permeation time is important for both permeation percentage and amount, that is, longer permeation time results into higher drug permeation. This is consistent with the results obtained in the Fick's model in Figure 5. In transdermal patch formulations, the amount of drug is generally provided in excess to create a concentration gradient against skin, to ensure drug permeation from the patch into skin. With excess amount of drug in the patch, more drug can permeate over extended permeation time.

In addition to permeation time, the contribution from other features varies. For permeation percentage, it was shown that larger MN surface area could result in higher drug permeation percentage (Figure 8). For permeation amount, it was found that greater drug loading in MN patch can result in more drug permeation through skin (Figure 8). Compared with permeation percentage, permeation amount is of greater importance, as it represents the dose to be delivered into human, which has always been the focus of transdermal drug delivery, that is, to enhance drug permeation through skin. On the other hand, permeation percentage represents the amount of drug being absorbed into human, as a percentage of total drug loading in the patch. Hence, it indicates the efficiency of the drug delivery system (bioavailability), and larger MN surface area is associated with greater bioavailability.

Apart from permeation time, MN surface area, and drug loading in MN, other features (drug MW, MN length, MN type, skin type), are of less relevance in the prediction, which seems to be contradicting the rules for drug permeation through skin. For example, the MW of the drug is a known parameter affecting drug permeation through skin, with lower MW being associated with higher drug permeation.^50^ This discrepancy may also be ascribed to the small number of data points in this study, which put too much weight on drug loading in MNs. Hence, more data need to be generated experimentally to increase the weights of the other parameters, such as MW of drugs and MN geometries.^51^

It was also noted that MN type was not a significant feature even though the two types of MNs (hydrogel and plastic) have distinct mode of action. For hydrogel MNs, drugs are impregnated inside the polymeric materials of the MN and are gradually released into skin tissue.^31^ For the plastic MNs, they are used to pre‐treat the skin before drugs, which are dissolved in an aqueous solution, are applied onto the pre‐treated skin.^9^ Fundamentally, however, both types of MN can enhance drug permeation by piercing skin to create multiple passages for drugs to permeate through. In this regard, they share the same mechanism, hence are not very different from each other after all.

For future studies, the model can be improved by using a larger data set, including different types and shapes of MN, various drugs, and different drug loading, to reduce the deviation between the predictions and the experimental data. The model is aimed at predicting the amount of drug which permeates skin after a certain period of MN patch application onto skin. Furthermore, the model is also expected to make predictions for drugs, which are not in the training data set. This will be useful to optimize the MN formulations for a given drug and can be used to predict the skin permeation profile of new drugs.

## 5 CONCLUSION

Four simulation models were established to predict drug permeation through skin. Among which the XGBoost model provided the best prediction results. The RF method ranked second in predicting drug permeation percentage, while Fick's law was in the second position in predicting drug permeation amounts. Taken together, machine learning methods were found useful in predicting drug permeation through skin treated with MNs.

## AUTHOR CONTRIBUTIONS

**Yunong Yuan:** Formal analysis (lead); investigation (lead); methodology (lead); software (lead); validation (equal); visualization (equal); writing – original draft (equal); writing – review and editing (equal). **Yiting Han:** Conceptualization (equal); data curation (equal); investigation (equal); methodology (equal); writing – review and editing (equal). **Chun Wei Yap:** Investigation (equal); methodology (equal); supervision (equal); writing – review and editing (equal). **Jaspreet S. Kochhar:** Data curation (equal); investigation (equal); methodology (equal); writing – review and editing (equal). **Hairui Li:** Data curation (equal); investigation (equal); methodology (equal); writing – review and editing (equal). **Xiaoqiang Xiang:** Conceptualization (equal); funding acquisition (equal); investigation (equal); methodology (equal); resources (equal); writing – review and editing (equal). **Lifeng Kang:** Conceptualization (equal); data curation (equal); formal analysis (equal); funding acquisition (equal); investigation (equal); methodology (equal); project administration (equal); resources (equal); supervision (equal); visualization (equal); writing – review and editing (equal).

## CONFLICT OF INTEREST STATEMENT

The authors declare no conflicts of interest.

### PEER REVIEW

The peer review history for this article is available at https://www.webofscience.com/api/gateway/wos/peer-review/10.1002/btm2.10512.

## ETHICS STATEMENT

The data used in this study are experimental results from the authors' previous studies with required ethical approvals for using animal and human skin tissue samples.

## Supporting information


**Data S1.** Supporting Information

**Data S2.** Supporting Information

**Figure S1.** New drug permeation amount prediction: (a) BSA; (b) GHK(H); (c) GHK(R); (d) rhodamine B(R); (e) lidocaine(H); (f) lidocaine(R); (g) caffeine(H); (h) Cu(R); and (i) Cu(H). R, in vitro rat skin permeation experiments; H: in vitro human skin permeation experiments.
**Figure S2.** New drug permeation percentage prediction: (a) BSA; (b) GHK(H); (c) GHK(R); (d) rhodamine B(R); (e) lidocaine(H); (f) lidocaine(R); (g) caffeine(H); (h) Cu(R); and (i) Cu(H). R, in vitro rat skin permeation experiments; H, in vitro human skin permeation experiments.

## Data Availability

The data are available as supplementary materials published together with this article.
