# Supplementary material for: Prediction of drug permeation through microneedled skin by machine learning
Source: Bioeng Transl Med. 2023 Apr 3;8(6):e10512. doi: 10.1002/btm2.10512 (PMC10658566; doi:10.1002/btm2.10512)
Supplement: Supplementary file 2 — Data S2. Supporting Information [file BTM2-8-e10512-s002.docx]

# **Prediction of drug permeation through microneedled skin by machine learning**

**Supplementary Information (SI) 2**

**R language for the 3 machine learning methods**

rm(list=ls())

library("xgboost")

library("Matrix")

library("randomForest")

set.seed(0)

train <- read.csv("train set.csv")

test <- read.csv("test set.csv")

train_withoutR <- train[,-8]

test_withoutR <- test [,-8]

#Multi-linear regression

lm<-lm(Results~. -1,data = train)

summary(lm)

lm_pred <- predict(lm, test)

output <- rbind(test$Releasing.time,lm_pred, test$Results)

write.csv(output,file="MLRoutput.csv")

#Random Forest

RForest<- randomForest(formula=Results~.-1,data=train,num.trees=500,mtry=6,importance=TRUE,proximity=TRUE)

plot(RForest)

RForest

importance(RForest)

varImpPlot(RForest)

RF_predict<-predict(RForest,test)

output <- rbind(test$Releasing.time,RF_predict, test$Results)

write.csv(output,file="RFoutput.csv")

#XgBoost

train_matrix <- sparse.model.matrix(Results ~ . - 1, data = train)

train_matrix

test_matrix <- sparse.model.matrix(Results ~ . - 1, data = test)

train_label<-train$Results

test_label<-test$Results

train_fin <- list(data = train_matrix,label=train_label)

test_fin <- list(data = test_matrix,label=test_label)

dtrain <- xgb.DMatrix(data = train_fin$data,label=train_fin$label)

dtest <- xgb.DMatrix(data = test_fin$data,label=test_fin$label)

xgb <- xgboost(data = dtrain, max_depth = 3, eta = 0.2, objective = "reg:squarederror",nround = 45)

pred<-predict(xgb,dtest)

error=var(test$Results-pred)

error

importance <- xgb.importance(train_matrix@Dimnames[[2]],model = xgb)

head(importance)

xgb.plot.importance(importance)

#xgb.plot.importance(importance_matrix = importance)

output <- rbind(test$Releasing.time,pred, test$Results)

write.csv(output,file="output.csv")

**C language for Fick’s law**

#include <stdio.h>

#include <math.h>

#include <stdlib.h>

int N_L, N_W

double dt=0.0001

double sum = 0

int main()

{

double L = 1000, W = 250

double l = 1000, w = 75

double D = 700

double N = 64

double m = 1637;

double V = 1/2.0*w*l;

double C0 = m / (V * N)

double C00 = 0

double dx = 2, dy = 2;

int i, j

int n = 0;

double t = 0

double T = 60 * 50

FILE *pf1 = NULL;

pf1 = fopen("Drug concentrtaion evolution.txt", "w");

if (pf1 == NULL)

{

printf("Open file error!\n");

return 0;

}

FILE *pf2 = NULL;

pf2 = fopen("results1.txt", "w");

if (pf2 == NULL)

{

printf("Open file error!\n");

return 0;

}

FILE *pf3 = NULL;

pf3 = fopen("results2.txt", "w");

if (pf3 == NULL)

{

printf("Open file error!\n");

return 0;

}

N_L = (int)(L / dx + 0.5) + 1;

N_W = (int)(W / dy + 0.5) + 1;

if (N_L < 1 || N_W < 1 )

{

printf("N_L, N_W are not natural number!/n");

return 0;

}

double **C;

double **C1;

C = (double * *)malloc(N_L * sizeof(double*));

C1 = (double * *)malloc(N_L * sizeof(double*));

for (i = 0; i < N_L; i++)

{

C[i] = (double *)malloc(N_W * sizeof(double));

C1[i] = (double *)malloc(N_W * sizeof(double));

}

if (C == NULL || *C == NULL)

{

fprintf(pf1, "C malloc error!/n");

return 0;

}

if (C1 == NULL || *C1 == NULL)

{

fprintf(pf1, "C1 malloc error!/n");

return 0;

}

for (i = 0; i < N_L; i++)

{

for (j = 0; j < N_W; j++)

{

if (i < -((l/dx) / (w/dy)) * j + (l/dx))

{

C[i][j] = C0;

}

else

{

C[i][j] = 0;

}

}

}

for (t = 0; t < T; t=t+dt,n++)

{

for (i = 0; i < N_L; i++)

{

for (j = 0; j < N_W; j++)

{

if (i == 0 && j == 0)

{

C1[i][j] = C[i][j] + dt*D * ((C[i + 1][j] - C[i][j]) / dx / dx + ((C[i][j + 1] - C[i][j]) / dy / dy));

}

else if (i == 0 && j == (N_W - 1))

{

C1[i][j] = C[i][j] + dt * D * ((C[i + 1][j] - C[i][j]) / dx / dx + ((C[i][j - 1] - C[i][j]) / dy / dy));

}

else if (i == (N_L - 1) && j == 0)

{

C1[i][j] = C[i][j] + dt * D * ((C[i - 1][j] - C[i][j]) / dx / dx + ((C[i][j + 1] - C[i][j]) / dy / dy) + ( - C[i][j]) / dx / dx);

}

else if (i == (N_L - 1) && j == (N_W - 1))

{

C1[i][j] = C[i][j] + dt * D * ((C[i - 1][j] - C[i][j]) / dx / dx + ((C[i][j - 1] - C[i][j]) / dy / dy) + ( - C[i][j]) / dx / dx);

}

else if (j == 0)

{

C1[i][j] = C[i][j] + dt * D * ((C[i + 1][j] + C[i - 1][j] - 2 * C[i][j]) / dx / dx + ((C[i][j + 1] - C[i][j]) / dy / dy));

}

else if (i == 0)

{

C1[i][j] = C[i][j] + dt * D * ((C[i + 1][j] - C[i][j]) / dx / dx + ((C[i][j - 1] + C[i][j + 1] - 2 * C[i][j]) / dy / dy));

}

else if (j == (N_W - 1))

{

C1[i][j] = C[i][j] + dt * D * ((C[i + 1][j] + C[i - 1][j] - 2 * C[i][j]) / dx / dx + ((C[i][j - 1] - C[i][j]) / dy / dy));

}

else if (i == (N_L - 1))

{

C1[i][j] = C[i][j] + dt * D * ((C[i - 1][j] - C[i][j]) / dx / dx + ((C[i][j + 1] + C[i][j - 1] - 2 * C[i][j]) / dy / dy) + ( - C[i][j]) / dx / dx);

}

else

{

C1[i][j] = C[i][j] + dt * D * ((C[i + 1][j] + C[i - 1][j] - 2 * C[i][j]) / dx / dx + ((C[i][j + 1] + C[i][j - 1] - 2 * C[i][j]) / dy / dy));

}

}

}

for (i = 0; i < N_L; i++)

{

for (j = 0; j < N_W; j++)

{

C[i][j] = C1[i][j];

}

}

for (j = 0; j < N_W; j++)

{

C00 = C00 + dt * D * (C[N_L - 1][j]) / dx / dx*dy;

}

if (n % 50000 == 0)

{

fprintf(pf2, "%lf\t%lf\n", t/60.0, C00 * 2 * N);

}

}

for (i = 0; i < N_L; i++)

{

free(C[i]);

free(C1[i]);

}

free(C);

free(C1);

fclose(pf1);

}
