# Supplementary material for: Prediction of drug permeation through microneedled skin by machine learning
Source: Bioeng Transl Med. 2023 Apr 3;8(6):e10512. doi: 10.1002/btm2.10512 (PMC10658566; doi:10.1002/btm2.10512)
Supplement: Supplementary file 3 — Figure S1. New drug permeation amount prediction: (a) BSA; (b) GHK(H); (c) GHK(R); (d) rhodamine B(R); (e) lidocaine(H); (f) lidocaine(R); (g) caffeine(H); (h) Cu(R); and (i) Cu(H). R, in vitro rat skin permeation experiments; H: in vitro human skin permeation experiments. Figure S2. New drug permeation percentage prediction: (a) BSA; (b) GHK(H); (c) GHK(R); (d) rhodamine B(R); (e) lidocaine(H); (f) lidocaine(R); (g) caffeine(H); (h) Cu(R); and (i) Cu(H). R, in vitro rat skin permeation experiments; H, in vitro human skin permeation experiments. [file BTM2-8-e10512-s003.docx]

# **Prediction of drug permeation through microneedled skin by machine learning**

**Supplementary information (SI) 3**


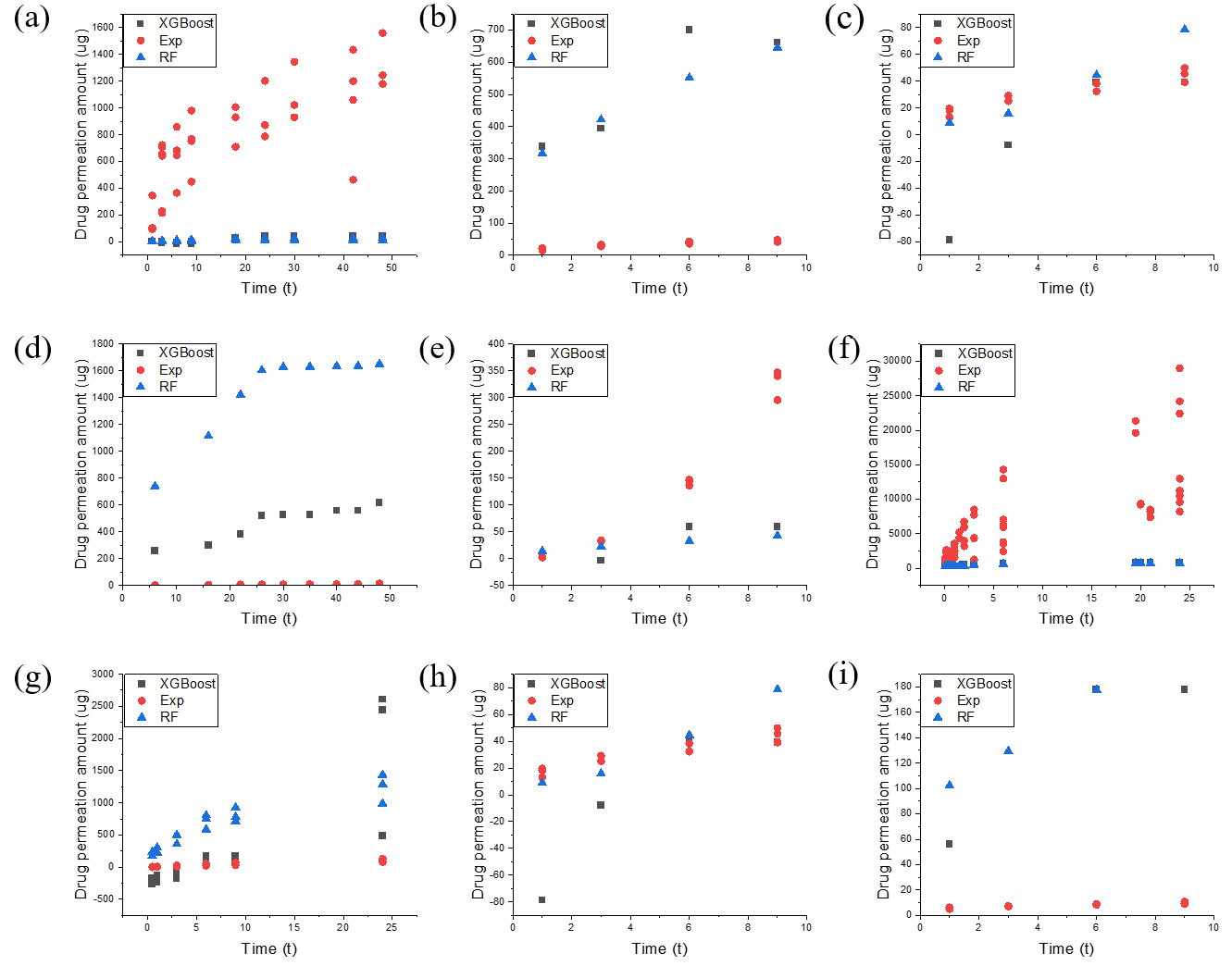


**Figure S1.** New drug permeation amount prediction: (a) BSA; (b) GHK(H); (c) GHK(R) (d) Rhodamine B(R); (e) Lidocaine(H); (f) Lidocaine(R); (g) Caffeine(H); (h) Cu(R); and (i) Cu(H). (R: in vitro rat skin permeation experiments, H: in vitro human skin permeation experiments


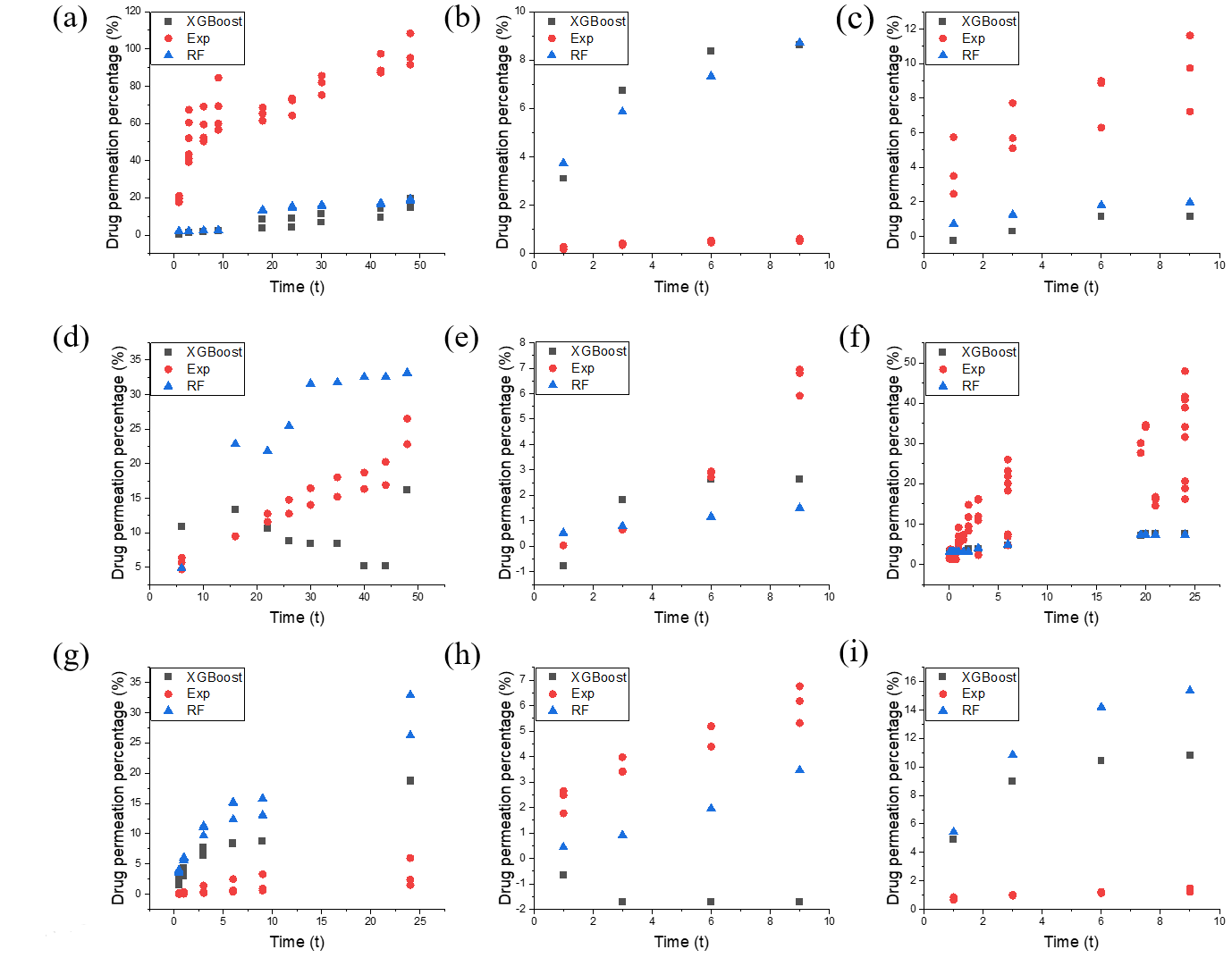


**Figure S2.** New drug permeation percentage prediction: (a) BSA; (b) GHK(H); (c) GHK(R) (d) Rhodamine B(R); (e) Lidocaine(H); (f) Lidocaine(R); (g) Caffeine(H); (h) Cu(R); and (i) Cu(H). (R: in vitro rat skin permeation experiments, H: in vitro human skin permeation experiments
